# Vaccine-Associated Proteinase 3 Anti-neutrophil Cytoplasmic Antibodies (PR3-ANCA) Vasculitis: A Case Report and Review of Emerging Evidence

**DOI:** 10.7759/cureus.88869

**Published:** 2025-07-27

**Authors:** Rhythm Shukla, Shivankur Singh, Huzefa Ansari

**Affiliations:** 1 Department of General Medicine, Dr. D. Y. Patil Medical College, Hospital and Research Centre, Dr. D. Y. Patil Vidyapeeth (Deemed to be University), Pune, IND; 2 Department of Cardiology, All India Institute of Medical Sciences, Bhopal, IND; 3 Department of General Medicine, Vardhman Mahavir Medical College and Safdarjung Hospital, New Delhi, IND

**Keywords:** anca, antineutrophil cytoplasmic antibody (anca)-associated vasculitis (aav), autoimmune flare-up, c-anca/proteinase 3 (pr3)-positive granulomatosis with polyangiitis, covid-19 vaccine, covid-19 vaccine complication, mpo-anca, pr3-anca, vaccine-induced vasculitis, wegener's granulomatosis

## Abstract

Antineutrophil cytoplasmic antibody (ANCA)-associated vasculitis (AAV) is a rare autoimmune condition characterized by necrotizing inflammation of small- to medium-sized vessels. While various environmental triggers have been implicated, the role of the COVID-19 vaccine as a possible trigger is still being studied.

We describe the case of a 21-year-old female patient who developed systemic inflammatory symptoms within 24 hours of receiving her second dose of the Covishield (ChAdOx1 nCoV-19) COVID-19 vaccine. Initial manifestations included acute-onset polyarthralgia with tenderness and fatigue, followed by multiple episodes of vomiting, nausea, and diffuse abdominal discomfort. The physical examination revealed a diffuse, non-blanching purpuric rash over the lower extremities. Laboratory evaluation revealed severe anemia, raised inflammatory markers, and impaired renal and hepatic function. Further tests revealed the rheumatoid (RA) factor being positive, followed by cytoplasmic ANCA (c-ANCA) proteinase 3 (PR3), strongly positive. Based on clinical and serological findings, a diagnosis of PR3-AAV was established. The patient received packed red blood cell transfusions, was initiated on high-dose corticosteroids, and was subsequently treated with rituximab. Clinical improvement was observed alongside normalization of inflammatory and renal parameters.

This case highlights a potential temporal association between COVID-19 vaccination and the onset of AAV. Although a direct cause cannot be established, awareness of this possible complication is essential for the timely recognition and management of post-vaccination autoimmune phenomena.

## Introduction

Antineutrophil cytoplasmic antibody (ANCA)-associated vasculitis (AAV) is a group of rare autoimmune disorders characterized by necrotizing inflammation of small- to medium-sized blood vessels. The major subtypes include granulomatosis with polyangiitis (GPA) associated with proteinase 3 (PR3-ANCA) and microscopic polyangiitis (MPA) typically associated with myeloperoxidase (MPO-ANCA) and eosinophilic granulomatosis with polyangiitis (eGPA), which may also be MPO-ANCA positive or ANCA-negative [1]. Clinical manifestations can range from constitutional symptoms and arthritis to life-threatening renal and pulmonary involvement [2].

The incidence of AAV is estimated at 0.5 to 2.6 per 100,000 individuals annually, with most cases occurring in middle-aged adults [3]. While the etiology remains incompletely understood, genetic predisposition and environmental triggers, including infections and medications, have been implicated [4].

Since the rollout of COVID-19 vaccination programs, rare cases of autoimmune phenomena, including AAV, have been reported following immunization. The temporal association between COVID-19 vaccines and AAV is not yet fully understood, but emerging case reports suggest a potential link, particularly in individuals with genetic susceptibility or pre-existing immune dysregulation [5-7].

We present the case of a 21-year-old female patient who developed PR3-AAV shortly after receiving her second dose of the Covishield (ChAdOx1 nCoV-19) COVID-19 vaccine. This report aims to contribute to the growing body of literature exploring post-vaccination autoimmune complications and to raise clinical awareness of this rare but potentially serious condition.

## Case presentation

A 21-year-old previously healthy female patient presented with multiple systemic symptoms that developed shortly after receiving her second dose of the Covishield vaccine. Her first dose of the COVID-19 vaccine was on 18^th^ July 2021, followed by a second dose on 14^th^ October 2021. Within 24 hours of vaccination, she reported fatigue, generalized malaise, and mild arthralgia. Over the following two weeks, her symptoms progressed to include severe joint pain, swelling of the left knee, and a non-blanching purpuric rash over the shins and dorsum of the feet. She also experienced multiple episodes of vomiting, nausea, and diffuse abdominal pain.

On physical examination, she appeared pale and fatigued. There was significant tenderness and swelling of the left knee joint and a non-blanching petechial rash was noted on the left lower limb. On ocular examination, episcleritis was noted. No lymphadenopathy or hepatosplenomegaly was observed. Cardiovascular, respiratory, and neurological examinations were unremarkable.

Initial laboratory investigations revealed normocytic normochromic anemia (hemoglobin 7.4 g/dL) and elevated inflammatory markers. Urinalysis showed microscopic hematuria. Autoimmune workup revealed a strongly positive cytoplasmic ANCA (c-ANCA) with a PR3 titer of 141.9 units/ml (Table 1).

**Table 1:** Laboratory investigation reports ELISA: enzyme-linked immunosorbent assay

Test	Result	Normal Range
C-reactive protein (CRP)	88.25 mg/L	0-6 mg/L
Erythrocyte sedimentation rate (ESR)	60 mm/hr	1–20 mm/hr
Hemoglobin	7.4 g/dL	12–15 g/dL
Red blood cell (RBC)	2.62 mill/cmm	4.0 - 5.0 mill/cmm
Packed cell volume (PCV)	23.10%	36–46%
Lymphocytes	15.60%	20.0 - 45.0%
Eosinophils	9.10%	1.0 - 6.0%
Absolute lymphocyte count	1480/cmm	1500.0 - 4000.0/cmm
Absolute eosinophil count	860/cmm	40.0 - 400.0/cmm
Serum iron	26 mcg%	50–170 mcg%
Ferritin	34.97 ng/mL	13–150 ng/mL
Serum total iron-binding capacity (TIBC)	260.5 mcg%	270.0 - 330.0 mcg%
Transferrin saturation	9.98%	16–140%
Estimated glomerular filtration rate (eGFR)	154.77 mL/min	>90 mL/min
Creatinine	0.53 mg%	0.57 - 1.11
Serum calcium	8.24 mg %	8.4 - 10.2
Urine RBCs	22/hpf	0–2/hpf
Total bilirubin	1.62mg%	> 5 days: 0 - 1.5 mg%
Direct bilirubin	0.72 mg%	< 0.3 mg%
Indirect bilirubin	0.9 mg %	Up to 0.6 mg%
Cytoplasmic antineutrophil cytoplasmic antibody (c-ANCA) (proteinase 3 (PR3))	141.9 units/mL	<20 units/mL (ELISA)
Anti-SARS-CoV-2 IgG	Positive	

**Figure 1:**
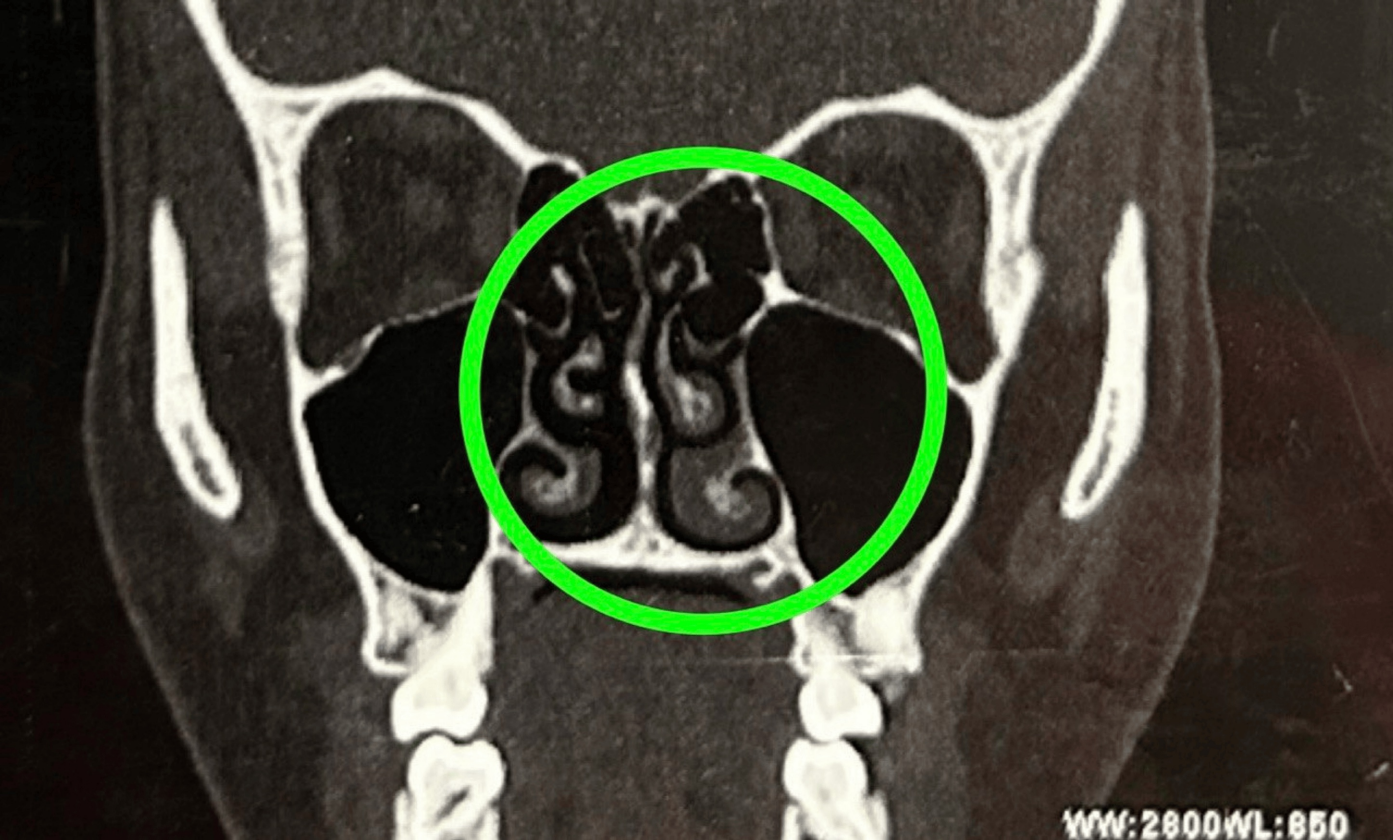
Mild mucosal hypertrophy involving the left middle and inferior turbinates

**Figure 2:**
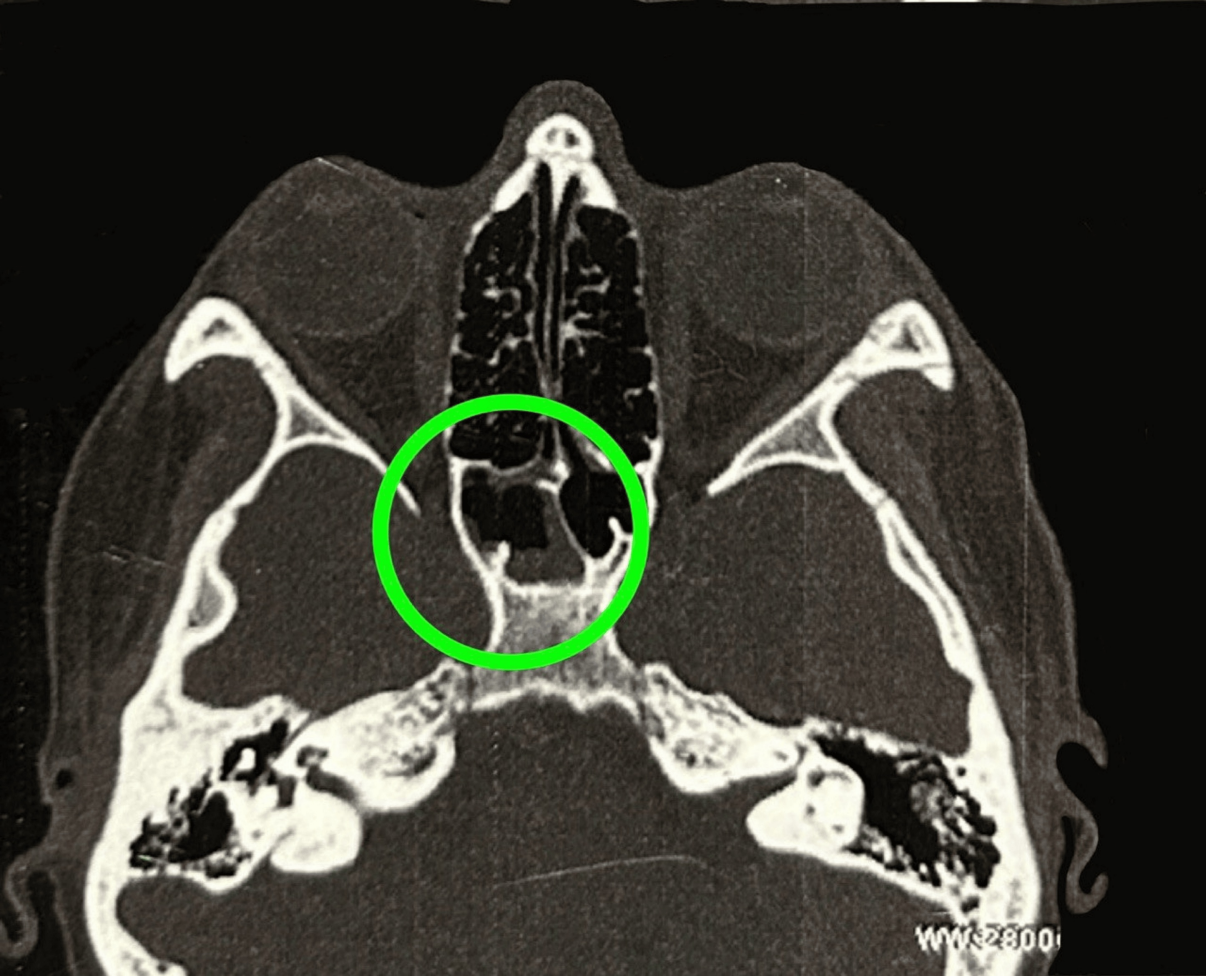
Mucosal thickening in the right sphenoid sinus

**Figure 3:**
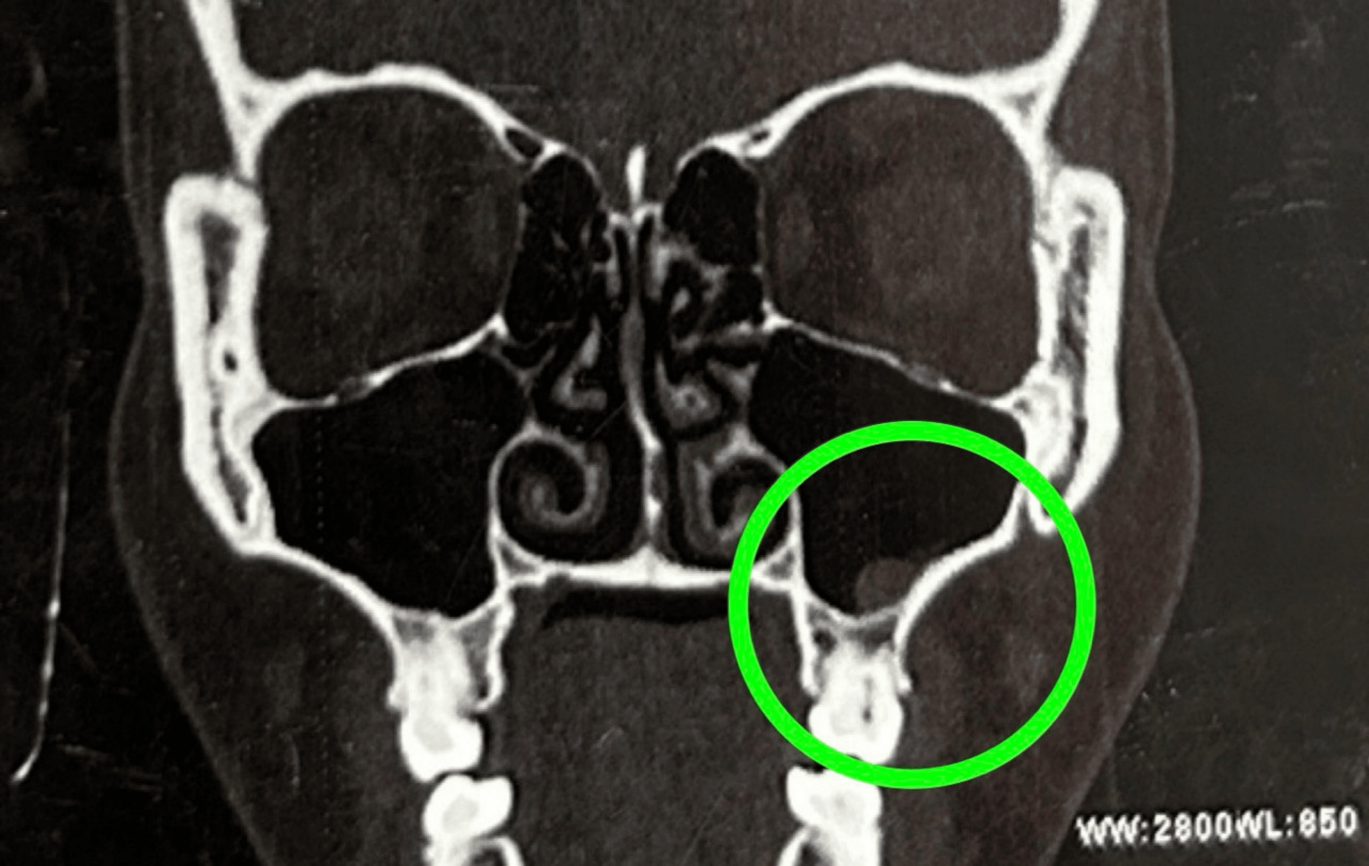
Polypoidal mucosal thickening along the floor of left maxillary sinus

A thorough abdominal ultrasound was performed as the initial diagnostic imaging, and it had no significant abnormalities. A computed tomography (CT) scan of the paranasal sinuses revealed mild mucosal hypertrophy involving the left middle and inferior turbinates (Figure 1), mucosal thickening in the right sphenoid sinus (Figure 2), and polypoidal mucosal thickening along the floor of the left maxillary sinus with mild deviation of the bony nasal septum towards the left (Figure 3). The concurrent chest X-ray was unremarkable. Renal biopsy was considered due to potential systemic involvement; however, the procedure was postponed due to clinical improvement and patient stabilization after intervention.

The patient was admitted and started on high-dose intravenous corticosteroids (methylprednisolone). She also received packed red blood cell transfusions for anemia. Due to the severity of symptoms and risk of organ involvement, rituximab therapy was initiated, followed by oral prednisolone and co-trimoxazole. Her clinical condition improved steadily over the next several weeks, with resolution of joint symptoms, rash, and gastrointestinal complaints. Follow-up laboratory testing showed normalization of inflammatory markers and gradual improvement in renal function.

## Discussion

AAV is an uncommon yet potentially severe autoimmune condition marked by necrotizing inflammation of small- to medium-sized blood vessels [1]. Clinically, it often manifests with renal impairment and systemic symptoms, including joint pain, purpuric skin rashes, respiratory complaints, and gastrointestinal disturbances [2]. PR3-ANCA positivity is typically associated with GPA, a major subtype of AAV [7].

In the present case, a previously healthy 21-year-old woman developed systemic symptoms, polyarthralgia, and a purpuric rash shortly after receiving her second dose of the Covishield vaccine. The constellation of her clinical presentation, along with elevated PR3-ANCA titers and evidence of renal involvement, was consistent with a diagnosis of AAV. The close temporal association between the vaccination and symptom onset suggests a possible immune-mediated response triggered by the vaccine.

Although definitive causality between COVID-19 vaccines and AAV still remains under study, several pathophysiological mechanisms have been proposed. One potential explanation is molecular mimicry, whereby vaccine antigens share structural similarities with self-antigens, inadvertently prompting an autoimmune response [8]. Another theory involves nonspecific immune activation through toll-like receptors or the effects of adjuvants, which may induce autoantibody production in genetically predisposed individuals [9]. Similar immune mechanisms have been implicated in autoimmune reactions following other vaccinations, including those for influenza [10].

A growing number of case reports have documented AAV following COVID-19 vaccination. For instance, Prabhahar et al. (2022) described a patient who developed PR3-ANCA vasculitis with renal involvement several weeks after receiving the Pfizer-BioNTech vaccine [11]. Similarly, Sekar et al. (2021) reported a case of MPO-ANCA vasculitis following the Moderna mRNA vaccine, further supporting a potential temporal association across different mRNA vaccine platforms [12]. Likewise, Uddin et al. (2022) reported a case of post-vaccination renal vasculitis accompanied by systemic inflammation [13]. These reports, along with our case, suggest a possible immune-mediated mechanism triggered by vaccination in susceptible individuals. The JAK-STAT signalling pathway has also been proposed as a potential contributor to vaccine-induced autoimmunity due to its role in immune regulation and cytokine signalling [14].

## Conclusions

This case describes the onset of PR3-AAV in a previously healthy young female following the second dose of the Covishield COVID-19 vaccine. The close temporal relationship between vaccination and symptom onset, including systemic features such as arthritis, petechial rash, abdominal symptoms, and renal involvement, along with a strongly positive c-ANCA (PR3 titer: 141.9 U/mL), suggests a possible immune-mediated trigger in a predisposed host. Early recognition and prompt immunosuppressive treatment led to clinical improvement, highlighting the importance of clinical vigilance in patients presenting with systemic symptoms after immunization. While causality cannot be established, this case adds to the growing body of literature on rare post-vaccination autoimmune phenomena and underscores the need for continuous post-marketing surveillance. 

Early diagnosis and treatment with immunosuppressants are important for the best results and are essential to prevent irreversible organ damage and improve clinical outcomes. It is important to emphasize that the benefits of COVID-19 vaccination continue to vastly outweigh the risks. This report is intended not to discourage vaccination but to highlight the importance of vigilance regarding potential autoimmune complications post-immunization. In conclusion, this case highlights the importance of post-immunization surveillance and investigation of uncommon autoimmune events, while reiterating that the overall benefit-risk ratio of COVID-19 vaccination continues to be overwhelmingly positive. 
